# Peptidorhamnomannan from *Lomentospora prolificans* modulates the inflammatory response in macrophages infected with *Candida albicans*

**DOI:** 10.1186/s12866-020-01931-3

**Published:** 2020-08-06

**Authors:** Mariana Ingrid Dutra da Silva Xisto, Suelen S. Santos, Luana Rossato, Fábio Seiti Yamada Yoshikawa, Rosa Maria Tavares Haido, Sandro Rogério de Almeida, Eliana Barreto-Bergter

**Affiliations:** 1 Departamento de Microbiologia Geral, Laboratório de Química Biológica de Microrganismos, Instituto de Microbiologia Paulo de Góes, Centro de Ciências da Saúde, Universidade Federal do Rio de Janeiro (UFRJ), Ilha do Fundão, Rio de Janeiro, RJ 21941-902 Brazil; 2 Departamento de Análises Clinicas e Toxicológicas, Faculdade de Ciências Farmacêuticas –USP, São Paulo, Brazil; 3 Departamento de Microbiologia e Parasitologia, Instituto Biomédico, UNIRIO, Rio de Janeiro, RJ Brazil

**Keywords:** Peptidorhamnomannan, *Lomentospora prolificans*, *Candida albicans*, Inflammatory response

## Abstract

**Background:**

Peptidorhamnomannan is a glycoconjugate that consists of a peptide chain substituted by *O*- and *N*-linked glycans, present on the cell surface of *Lomentospora prolificans*, a saprophytic fungus which is widely distributed in regions with temperate climates. *O*-linked oligosaccharides from peptidorhamnomannan isolated from *Lomentospora prolificans* conidia are recognized by macrophages mediating macrophage - conidia interaction. In this work, peptidorhamnomannan was isolated from *L. prolificans* mycelium cell wall and its role in macrophage - *Candida albicans* interaction was evaluated.

**Results:**

Purified peptidorhamnomannan inhibits the reactivity of rabbit immune sera to mycelial and conidia forms of *L. prolificans*, indicating that this glycoconjugate is exposed on the fungal surface and can mediate interaction with host immune cells. We demonstrated that peptidorhamnomannan leads to TNF-α production in J774 macrophages for 1, 2 and 3 h of incubation, suggesting that this glycoconjugate may have a beneficial role in the response to fungal infections. In order to confirm this possibility, the effect of peptidorhamnomannan on the macrophage - *C. albicans* interaction was evaluated. Macrophages treated with peptidorhamnomannan led to a lower fungal survival, suggesting that peptidorhamnomannan induces an increased fungicidal activity in macrophages. Furthermore, TNF-α levels were measured in supernatants after macrophage - *C. albicans* interaction for 1, 2 and 3 h. Peptidorhamnomannan treatment led to a higher TNF-α production at the beginning of the interaction. However, the release of TNF-α was not maintained after 1 h of incubation. Besides, peptidorhamnomannan did not show any inhibitory or fungicidal effect in *C. albicans* when used at 100 μg/ml but it was able to kill *C. albicans* at a concentration of 400 μg/ml.

**Conclusion:**

We suggest that peptidorhamnomannan acts as a molecular pattern on the invading pathogen, promotes TNF-α production and, thus, increases macrophage fungicidal activity against *Candida albicans*.

## Background

*Lomentospora prolificans* (formally *Scedosporium prolificans*) is an emerging opportunistic fungal pathogen causing localized and disseminated infections in immunocompetent and immunocompromised patients, respectively [1]. *L. prolificans* is a saprophytic fungus occurring worldwide in soil, sewage and polluted waters, and its occurrence is associated with human activity [2, 3]. The most important feature of *L. prolificans* is its inherent resistance to all currently available antifungal compounds, showing very low susceptibility and, as a consequence, infections caused by this species are associated with high morbidity and mortality rates [4]. *N*- and *O*-linked peptidorhamnomannans (PRM) were isolated from conidia and mycelium of *L. prolificans* and their *O*-linked oligosaccharides were identified by a combination of techniques including gas chromatography, mass spectrometry and nuclear magnetic resonance [5, 6]. Although *O*-linked oligosaccharides of PRM from *L. prolificans* conidia and mycelium share similar structures, PRM isolated from conidia has a 2-*O*-methyl rhamnose capping group in its *O*-linked oligosaccharides and has no β-Gal*p* side-chain in its hexasaccharide [5, 6]. Previous work from our group showed that *O*-glycosylation of PRM from conidia plays a role in the recognition and uptake of *L. prolificans* conidia by macrophages, killing of macrophages and production of pro-inflammatory cytokines [7]. However, the function of PRM from *L. prolificans* mycelium is not completely elucidated. The ability of mycelium PRM to protect mice against *L. prolificans* infection was investigated and the results showed that this glycoconjugate exacerbated the infection process by reducing the inflammatory response and facilitating the colonization, virulence and dissemination of the fungus [8]. Based on these results, we decided to investigate the effect of PRM isolated from *L. prolificans* mycelium on its ability to induce proinflammatory response in macrophages using *Candida albicans* as model fungus.

## Results

**Fig. 1:**
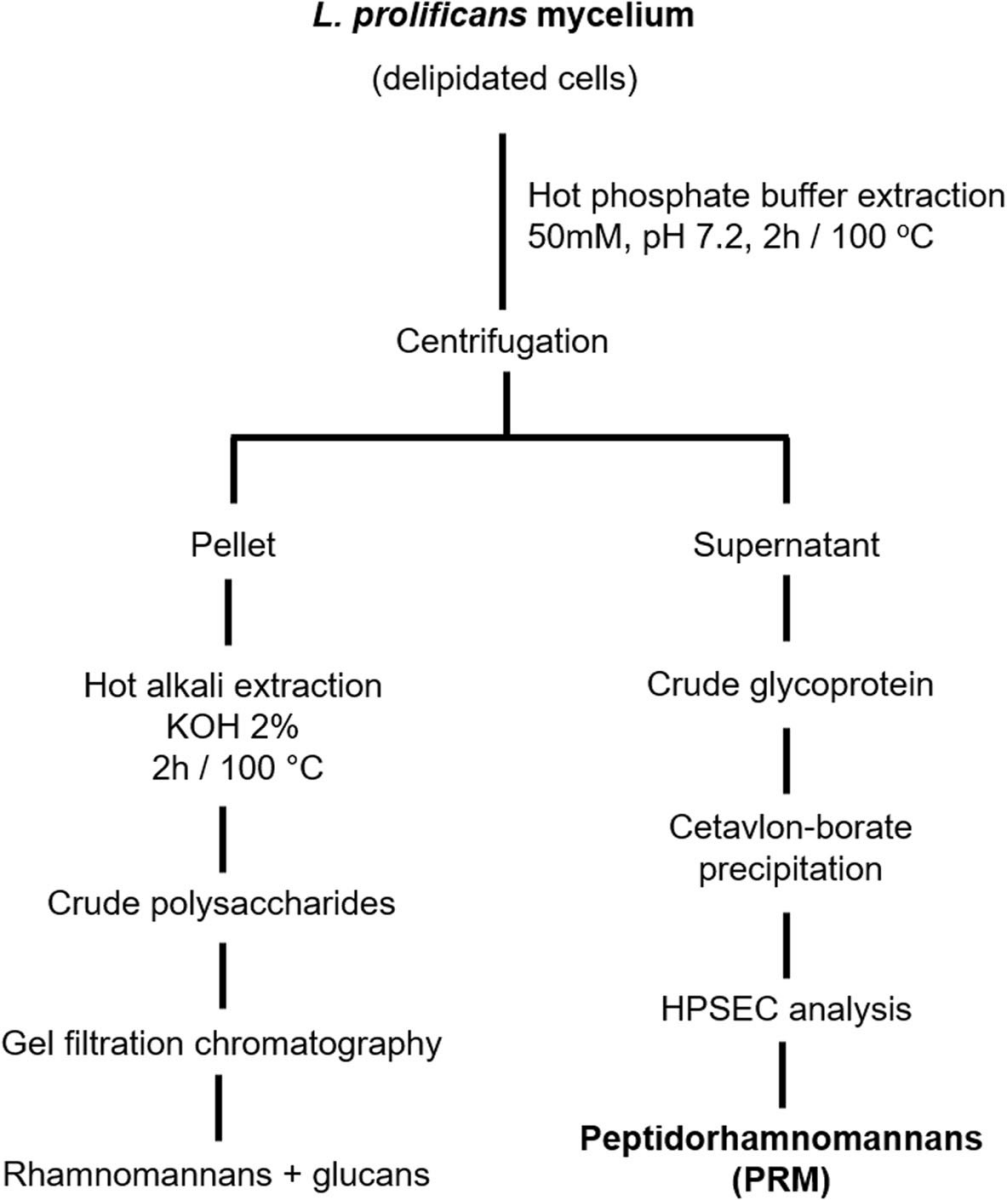
Extraction and purification scheme for peptidorhamnomannan from *L. prolificans* cell wall according to Barreto-Bergter et al. [5] and Figueiredo et al. [9]

The peptidorhamnomannan (PRM) used in the present study containing neutral carbohydrate (62%) and protein (35%) was isolated from *Lomentospora prolificans* mycelium (Fig. 1) and its structure was identified and characterized by Barreto-Bergter and colleagues [5].

### PRM is distributed on the surface of Lomentospora prolificans mycelium

**Fig. 2:**
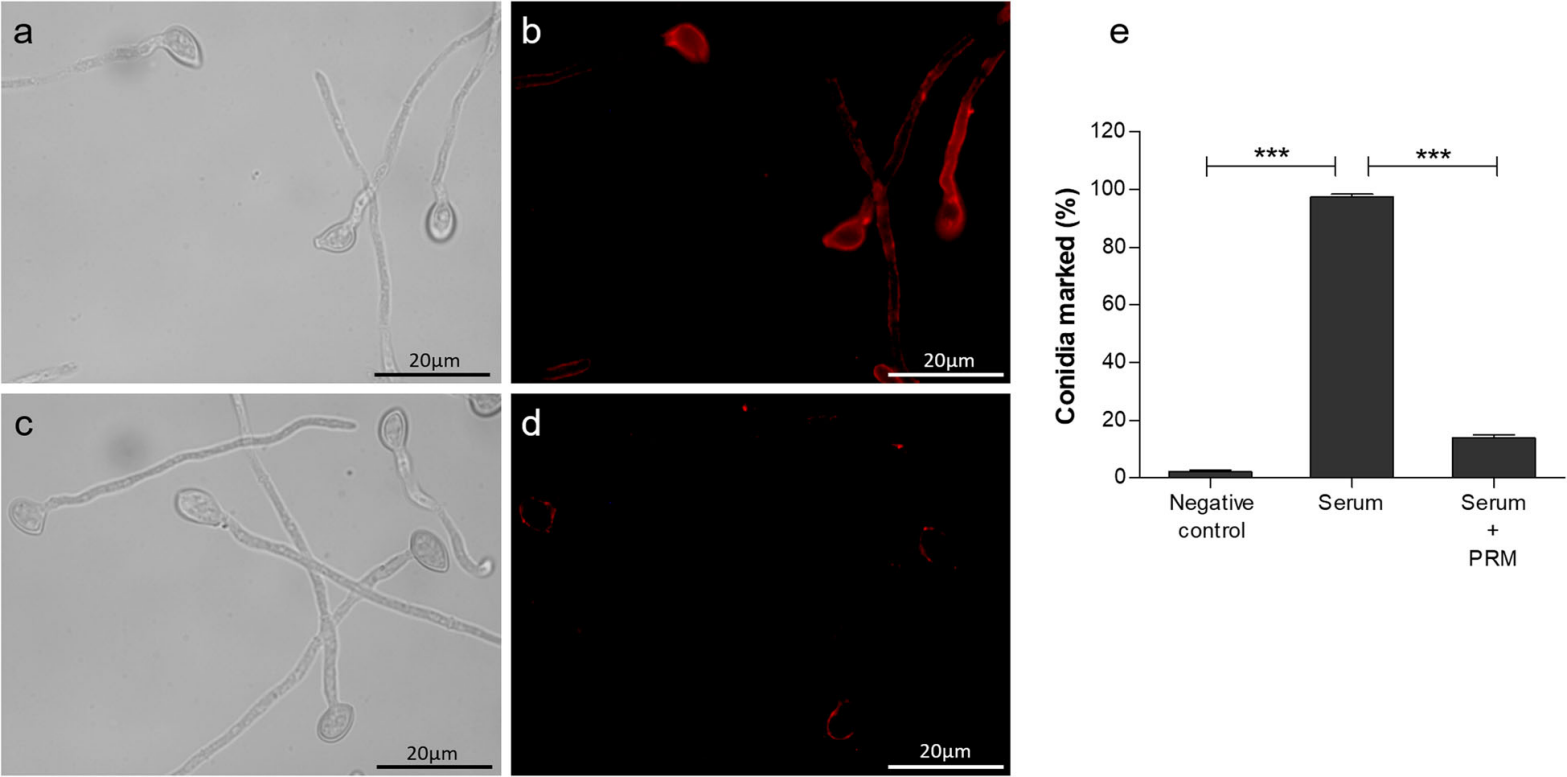
PRM is located on the fungal cell surface. Immune serum was able to bind to *L. prolificans* mycelial and conidial forms (**a** and **b**), but pre-incubation with soluble PRM was able to inhibit the binding between *L. prolificans* and immune serum (**c** and **d**), as observed by immunofluorescence microscopy. Similar data was observed by flow cytometry showing that conidia fluorescence is practically abolished by pre- treatment with PRM (**e**). Bar: 20 μm

To determine whether PRM is exposed on the surface of *L. prolificans* mycelia, rabbit immune serum raised against whole *L. prolificans* cells was employed in immunofluorescence assays. As demonstrated by fluorescence microscopy, the immune serum was able to recognize mycelium forms (Fig. 2a, b), whereas its reactivity was inhibited when serum was previously treated with soluble PRM (100 μg/ml) (Fig. 2c, d). In addition, flow cytometry showed that conidium fluorescence is practically abolished when the serum was pre-incubated with PRM purified from mycelium, confirming the expression of PRM on the surface of both, conidia and mycelium (Fig. 2e).

### PRM promotes TNF-α production by J774 macrophages

PRM from *L. prolificans* conidia is known to induce TNF-α secretion by peritoneal macrophages, and the *O*-linked oligosaccharidic chains are important moieties involved in this secretion [7]. Significant structural differences between PRM isolated from *L. prolificans* conidia and mycelium forms have been described [5, 6]. Therefore, we decided to evaluate the ability of mycelium derived PRM to induce TNF-α secretion using the macrophage-like cell line J774.

**Fig. 3:**
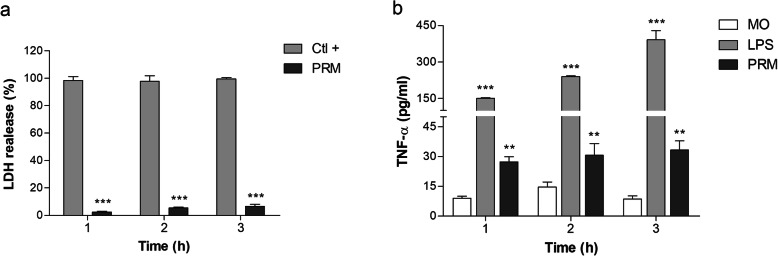
PRM induces TNF-α production in macrophages. **a**. Cytotoxicity assay of PRM at 100 μg/ml on J774 macrophages assessed by LDH activity for 1, 2, and 3 h. Ctl + positive control: maximal cell lysis induced by addition of a detergent. **b**. J774 macrophages were incubated with PRM (100 μg/ml) for 1, 2 and 3 h and cytokines were determined in the culture supernatants. MO: macrophage only. Values represent the mean ± SD of three independent experiments performed in triplicate. Asterisks denote values statistically different from control. *p < 0.05; **p < 0.01

The cytotoxicity of PRM was assessed by LDH activity at 100 μg/ml, and it was observed that PRM did not alter J774 macrophages viability (Fig. 3a). J774 macrophages were incubated with PRM at 100 μg/ml for 1, 2 and 3 h at 37 °C, and LPS at 1 μg/ml was used as positive control. The culture supernatant was collected and the concentration of TNF-α was measured. The results indicated that PRM was able to induce the release of TNF-α by J774 macrophages in the time intervals tested (Fig. 3b).

### PRM effect on macrophage – *Candida albicans* interaction

**Fig. 4:**
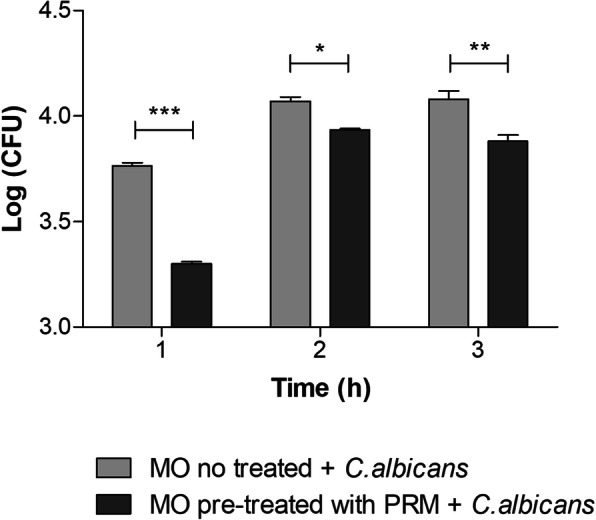
PRM promotes more efficient fungal clearance. J774 macrophages were incubated with PRM at 100 μg/ml for 1 h. After this incubation, PRM was removed and the monolayers were washed, and incubated with *C. albicans* yeast cells (1:1 ratio) for 1, 2 and 3 h. Fungi recovered from macrophages were determined by CFU assay. MO: Macrophages. Values represent the mean log ± SD of three independent experiments performed in triplicate. Asterisks denote values statistically different from control. *p < 0.05; **p < 0.01; ***p < 0.001

In order to analyze the ability of PRM to increase the microbicidal effect of macrophages against *C. albicans*, a common fungal pathogen in humans, J774 macrophages were incubated with PRM (100 μg/ml) for 1 h at 37 °C. After this period, the macrophage monolayer was rinsed with RPMI and *C. albicans* yeasts were added to the monolayer and incubated for 1, 2 and 3 h at 37 °C. PRM was able to lower the fungal burden on J774 macrophages, showing an increase of the microbicidal activity of the macrophage in the presence of PRM at all times tested (Fig. 4). Light micrographs are shown in Additional file 1.

### PRM induces an earlier TNF-α production wave in response to *C. albicans*

**Fig. 5:**
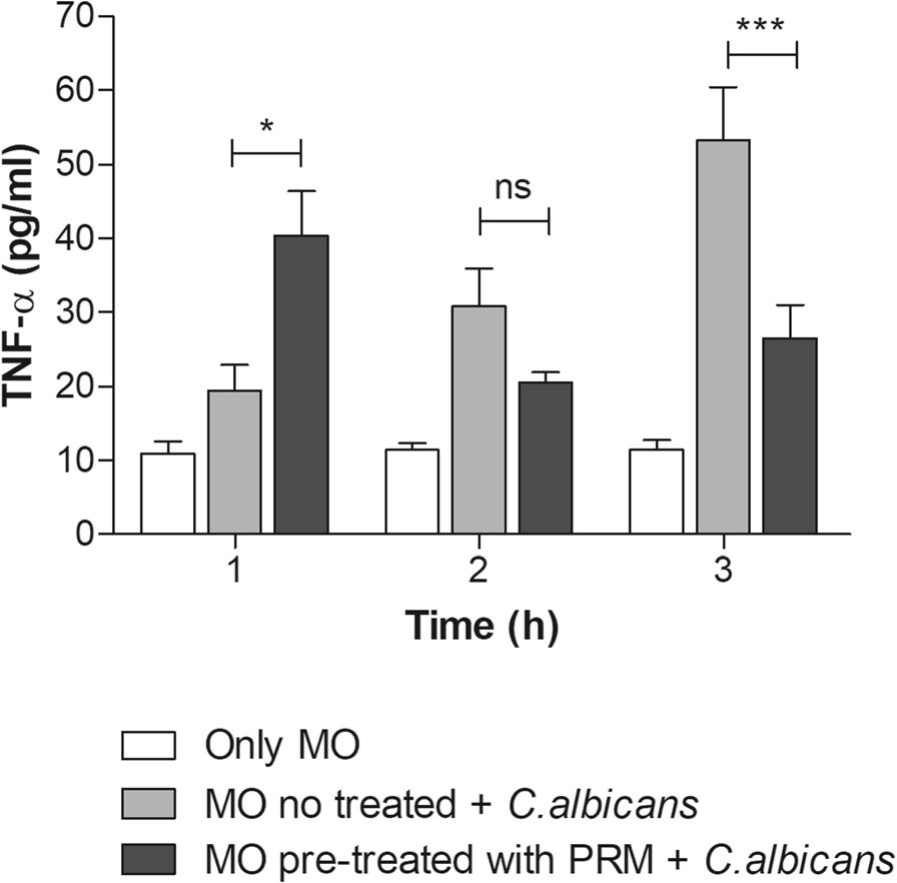
PRM induces an earlier TNF-α production wave in response to *Candida albicans*. J774 macrophages were incubated with PRM at 100 μg/ml for 1 h. After removal of PRM the monolayers were washed and incubated with *C. albicans* yeast cells (1:1 ratio) for 1, 2 and 3 h. Cytokines were determined in the culture supernatants. MO: Macrophages. Values represent the mean ± SD of three independent experiments performed in triplicate. Asterisks denote values statistically different from control. *p < 0.05; ***p < 0.001

PRM treatment led to a lower fungal survival, suggesting that macrophages exposed to these compounds show an increased fungicidal activity. In order to provide an explanation for these results, TNF-α levels were evaluated in the supernatants of macrophage cultured in the presence of *C. albicans* (Fig. 5). A higher TNF-α production at the beginning of the interaction was detected after PRM pre-treatment (1 h), as compared with the control without treatment. However, the release of TNF-α was not maintained after 1 h of incubation.

### PRM effect on *Candida albicans* viability

**Fig. 6:**
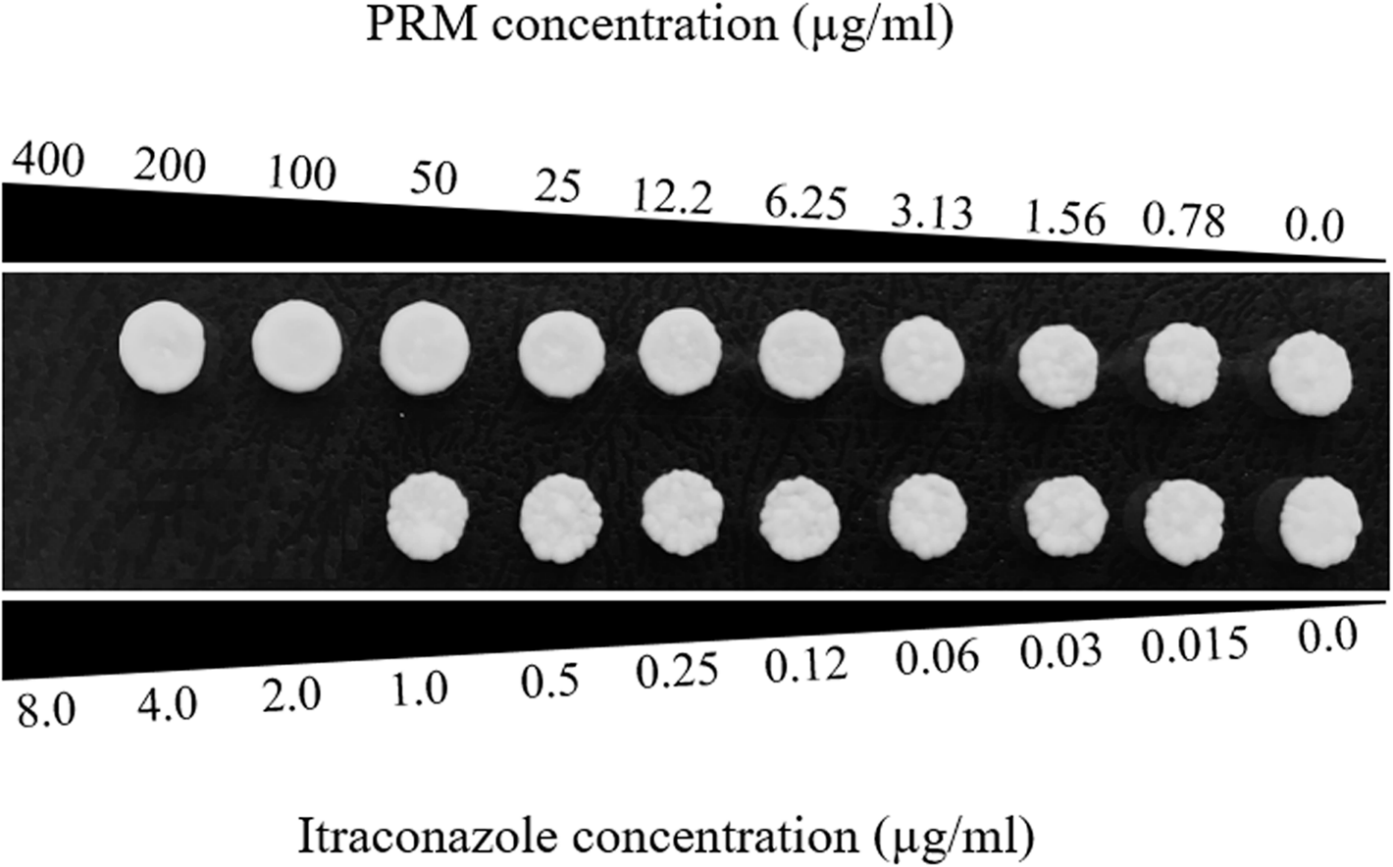
Growth of *C. albicans* in the presence of different concentrations (0.78–400 μg/ml) of PRM isolated from *L. prolificans* or in the presence of itraconazole (0.015–8 μg/ml) as positive control. Minimal fungicidal concentration (MFC) was determined as the first concentration in which fungal growth was not observed

In order to determine if the PRM concentrations used in the present work could have some effect on *C. albicans* viability, the Minimum Inhibitory Concentration (MIC) and Minimum Fungicidal Concentration (MFC) values of PRM were determined against *C. albicans*. As shown in Fig. 5, the PRM concentration of 100 μg/ml used in all experiments does not have any effect on *C. albicans.* PRM showed inhibitory and fungicidal activity only at 400 μg/ml (Fig. 6).

## Discussion

Peptidorhamnomanans are the main glycoconjugates present on the surface of the fungal cell wall of the *Scedosporium / Lomentospora* complex [10]. Although there are differences in the structure of the *O*-linked oligosaccharides, PRM from *L. prolificans*, *S. apiospermum* and *S. boydii* have a conserved “core” of α-Rha*p*-(1 → 3)-α-Man*p*-(1 → 2)-α-Man*p*-(1→ [10]. Differences in *O*-linked oligosaccharide structures suggest that they can be used as a potential antigens to diagnose infections caused by these fungi [11]. PRM is also involved in the interaction between fungal cells and epithelial cells (HEp2) or peritoneal macrophages [7, 12]. Recently, our group reported the importance of *O-*linked oligosaccharides from PRM isolated from *L. prolificans* conidia in inflammatory response through the induction of TNF-α secretion by mouse peritoneal macrophages [7]. Two predominant *O*-linked oligosaccharides with 2MeRha capping groups were identified after β-elimination of PRM and BioGel P-2 chromatography. One of them lack β-Gal*p* non-reducing end units [6].

In this work, we demonstrated that PRM from mycelium was able to inhibit the reactivity of rabbit immune sera with *L. prolificans* mycelial and conidia forms, indicating that PRM isolated from mycelium is exposed on surface of both *L. prolificans* forms. Besides minor structural differences in carbohydrate portions between mycelial and conidia forms of *L. prolificans* that were detected and mentioned above, an α-Rha*p* (1 → 3)-α-Man*p*-(1 → 2)-α-Man*p*-(1 → structural component is conserved and could explain the serum reactivity. Xisto et al. [7] observed similar results when they used PRM from *L. prolificans* conidia form, showing that PRM is present on the surface of *L. prolificans* conidia and can mediate fungal interactions with immune cells.

In this work, we have demonstrated that J774 macrophages secreted TNF-α in response to PRM isolated from mycelium after different incubation times as already observed by Xisto and colleagues [7] when they used PRM isolated from conidia, confirming that PRM has a pro-inflammatory activity and it is able to induce TNF-α even after shorter periods of incubation. *L. prolificans* PRM from mycelium and conidia plays a role in the proinflammatory cytokine induction, acting as Pathogen Associated Molecular Patterns (PAMPs) and, therefore, can be recognized by Pattern Recognition Receptor (PRRs).

Devillers et al. [13] showed that phospholipomannans from *C. albicans* can induce TNF-α production in macrophages and that this activity relies on the sugar portion. Carbohydrates are sensed by a large number of receptors, such as TLR2 (teichoic acid), TLR4 (LPS) and NOD1/NOD2 (peptidoglycan), but the classical receptors for sugars belong to the C-type lectin receptors family (CLRs) [14]. Among the CLRs, dectin-2 could have a prominent role in our case, since it recognizes *α*-mannans whose structures are found in PRM [5]. Xisto et al. [7] showed that conidial PRM triggered TNF-α release by macrophages and chemical removal of *O*-linked oligosaccharides from PRM abolished cytokine induction, suggesting that the *O*-linked oligosaccharidic chains are important moieties involved in inflammatory responses. Removal of *O*-linked oligosaccharides from *C. parapsilosis* cell wall mannoprotein by *β*-elimination affects the ability of *C. parapsilosis* to stimulate cytokine production by human PBMCs [15].

The production of TNF-α by macrophages is crucial in the defense against intracellular microorganisms [16]. The pro-inflammatory activity of PRM could have a beneficial role in the response to pathogens, since PRM increases TNF-α secretion by macrophages in the presence of *C. albicans* yeast compared with untreated macrophages. Besides, PRM treatment led to a higher TNF-α production at the beginning of the interaction, showing that macrophages pre-treated with PRM release TNF-α faster than macrophages without any treatment. At the beginning of macrophage - *C. albicans* interaction using macrophages pre-treated with PRM at 100 μg/ml, the increase of TNF-α production coincides with the lower fungal burden on macrophages. After 3 h of incubation the macrophage seems to succumb yeast filamentation to hyphae after being phagocytosed by macrophages (Additional file 1). However, macrophages pre-incubated with PRM seems to be more resistant in all times of incubation, and yeast seems to be less germinated after 3 h of incubation (Additional file 1). Furthermore, PRM at this concentration does not affect *C. albicans* viability, whereas higher concentrations (400 μg/ml) killed *C. albicans* in MIC and MFC assays.

In this work, the *L. prolificans* PRM induces TNF-α secretion constantly during the three incubation times (1, 2 and 3 h). However, the PRM stimulus was removed before the yeast addition, which allowed the activated macrophages interact only with *C. albicans* with no influence of PRM. At the first hour of incubation, these macrophages already activated by PRM and after interacting with others cell wall components from *C. albicans*, reach the peak of TNF-α release. After 2 and 3 h of incubation, the efficiency of treated macrophages to kill *C. albicans* decreased, and this may be related to a concomitant decrease in the TNF-α release by these macrophages. Geraldino et al. [16] showed that the pre-activation of macrophages with concanavalin-A leads to increased expression of mannose receptors which increase the internalization and death of *C. albicans* yeasts. The increase in the expression of these receptors is related to the increase in the TNF-α release, which contributes to a better clearance of internalized *C. albicans*. In the present study, the decrease of TNF-α release after 2 and 3 h of incubation with *C. albicans* allows a favorable environment for morphogenic switching from yeast to hyphal forms that could be leading to apoptosis [16–18]. In addition, these macrophages could be undergoing apoptosis due to hyper activation (first with PRM and later with *C. albicans*), which would also lead to decreased TNF-α release. Therefore, we suggest that PRM, acting as a PAMP, promotes TNF-α production.

*L. prolificans* PRM is a potent TNF-α inducer in peritoneal macrophages after 18 h of incubation**,** stimulating TNF-α release in similar concentrations to LPS [7]. However, in the present study, the TNF-α secretion induced by PRM was quantified in 1, 2 and 3 h of incubation and the amount of TNF-α secretion differs from vehicle control (RPMI medium), but the macrophage activation observed was lower compared with the LPS induction. Therefore, we concluded that the production of TNF-α was mediated by PRM and not by vehicle. We also concluded that the low production of TNF-α could be due to short incubation time tested in this work. Although production induced by PRM is lower when compared to LPS, the TNF-α produced was sufficient to induce the death of *C. albicans*.

Cytokine production in turn activates macrophages and increases their fungicidal activity. In *S. boydii* - macrophage interaction TNF-α release is mediated by TLR2 and TLR4 [9]. An *α*-glucan from *S. boydii* also induces TNF-α release by macrophages through TLR2 and CD14 [19]. *S. boydii* conidia possess a large number of rhamnomannans on the cell surface [10] and are recognized by TLR4 and CD14, and *S. boydii*-derived rhamnomannans were shown to be molecular patterns recognized by TLR4 [20]. These results showed the role of mannan-containing polymers in innate recognition of fungal pathogens.

## Conclusions

We demonstrated that PRM was able to induce TNF-α release by macrophages showing a pro-inflammatory activity. The pre-treatment of macrophages with PRM increased the macrophage fungicidal activity against *C. albicans* and resulted in a lower fungal burden in the macrophage. This increased TNF-α release at the beginning of the macrophage – *C. albicans* interaction seems to be important to promote a better fungal clearance by macrophages.

## Methods

### Microorganisms and growth conditions

A culture of *Lomentospora prolificans* strain FMR3569 was supplied by Dr. J. Guarro, Unitat de Microbiologia, Facultat de Medicina e Institut d’Estudis Avançats, Réus, Spain. It was grown in Erlenmeyer flasks containing 200 ml of modified Sabouraud medium, consisting of peptone (10 g/l), yeast extract (5 g/l) and glucose (40 g/l). Cultures were incubated at room temperature for 7 days with shaking (pre-inoculum). Cultures were diluted in 3 l of the same medium and incubated for another 7 days with shaking. Mycelia were filtered, washed with distilled water, and stored at − 20 °C. Conidia were grown at room temperature on Petri dishes containing modified Sabouraud agar medium. After 7 days, conidia were obtained by washing the agar surface with phosphate-buffered saline (PBS) and hyphal fragments and debris were removed by filtration through gauze.

*Candida albicans* (American Type Culture Collection - ATCC 90028) was maintained in Sabouraud Dextrose Agar (BD) at room temperature. A culture of 24- to 48 h old yeast cells growing in Sabouraud Dextrose Agar were used to prepare suspensions in cell culture media to be used in interaction assays with J774 macrophages.

### Extraction and purification of peptidorhamnomannans (PRM)

The crude glycoprotein was extracted from *L. prolificans* with 0.05 M phosphate buffer, pH 7.2, at 100 °C for 2 h and purified by hexadecyltrimethylammonium bromide (Cetavlon, Merck, Darmstadt, Germany) fractionation. The mother liquors from Cetavlon precipitation were adjusted to pH 8.8 in the presence of borate and the resulting precipitates recovered by centrifugation to give the major PRM fraction. This fraction was submitted to HPSEC analysis according to what was described by Barreto-Bergter et al. [5]. The extraction and purification scheme are depicted in Fig. 1.

### Rabbit immune sera

The rabbit immune serum against *L. prolificans* was supplied by R. M. T. Haido, Departamento de Microbiologia e Parasitologia, Instituto Biomédico da Universidade Federal do Estado do Rio de Janeiro, Brazil. The rabbit immune serum was obtained by inoculating white male rabbits with freeze-dried whole cells of *L. prolificans* (2 mg/ml dry weight) emulsified in an equal volume of complete Freund’s adjuvant; 1 ml of emulsion was injected intradermally at weekly intervals for 3 weeks [7, 21]. For an additional week, the same concentration was used in three intravenous injections at 2 days intervals. The hyperimmune serum obtained was used in flow cytometry and immunofluorescence experiments. Pre-immune serum was taken as a control.

### PRM immunolocalization on the *L. prolificans* surface - immunofluorescence

Freshly harvested mycelium was attached to coverslips coated with poly-L-lysine and then fixed in 1% paraformaldehyde in PBS for 1 h. After washing 3 times with PBS, nonspecific sites were blocked in blocking buffer (PBS-1% BSA) for a period of 1 h at 37 °C. The mycelium was incubated with rabbit anti-*L. prolificans* serum (1:50 dilution) overnight at 4 °C. After washing in PBS, anti-rabbit IgG conjugated with AlexaFluor (1:200) was added and incubated overnight at 4 °C. For the inhibition assays, the rabbit anti-*L. prolificans* serum diluted 1:50 was pre-incubated for 1 h at 37 °C with *L. prolificans* PRM (100 μg/ml) prior to the incubation with *L. prolificans* mycelium. To mount the glass slides, n-propyl gallate (VETEC) was used to preserve the sample and coverslips were sealed with nail polish. The recognition of PRM by antibody was visualized through Axioplan fluorescence microscopy [7].

### Flow cytometry

*L. prolificans* conidia fixed in 1% paraformaldehyde were incubated with rabbit anti-*L. prolificans* serum (1:50). For the inhibition assays, rabbit anti-*L. prolificans* serum diluted 1:50 was pre-incubated for 1 h at 37 °C with *L. prolificans* PRM (100 μg/ml) prior to incubation with *L. prolificans* conidia. The binding between *L. prolificans* conidia cells and immune serum was analyzed on a FACSCalibur flow cytometer (Becton Dickinson). Data from each experiment were analyzed using “Windows Multiple Document Interface Flow Cytometry Application (WinMDI) version 2.8 software”. Controls using only spores or the secondary antibody and spores were used. All procedures were carried out according to Xisto and colleagues [7].

### Cells lines

The J774 macrophage cell line was obtained from the ATCC. Cells were maintained in RPMI-1640 (Sigma-Aldrich) medium containing 10% Fetal Bovine Serum (Vitrocell), at 37 °C in 5% CO_2_.

### Cell viability of J774 macrophages

Macrophages were plated and after adhesion incubated with PRM (100 μg/ml) for 1, 2 and 3 h. After each incubation time, macrophage viability was assessed by measuring the release of the enzyme lactate dehydrogenase (LDH) by CytoTox assay (Promega) according to the manufacturer’s instructions. LDH activity at basal levels (without addition of fungal cells) and maximal cell lysis (induced by addition of 0.1% Triton X-100) was also determined as controls. LDH activity was calculated according to Yoshikawa et al. [22].

### J774 macrophage stimulation with PRM

J774 macrophages were plated in 24-wells plates (2.0 × 10^5^ cells/ml/well) and after adhesion, stimulated in RPMI medium with addition of *L. prolificans* PRM (100 μg/ml) or LPS (O111:B4, 1 μg/ml). After stimulation for 1, 2 or 3 h, the supernatants were recovered for TNF-α determination by ELISA (R&D Systems’s DuoSet kit) according to the manufacturer’s instructions. In order to rule out the possibility that the stimulating activity was due to contaminating lipopolysaccharides, polymyxin B (10 μg/ml) was added 5 min prior to the addition of the stimulus. After incubation, supernatants were harvested, centrifuged at 12000 rpm for 10 min to remove cell debris and immediately measured by ELISA.

### Macrophage effector functions – fungal viability

Fungal viability was assessed by determining the colony-forming units (CFU). J774 macrophages were plated in 24-well plates (2.0 × 10^5^ cells/ml/well) without glass slides, as described above. After each time of incubation, the culture was washed twice with PBS (Na_2_HPO_4_ 18 mM; NaH_2_PO_4_.H_2_O 3 mM and NaCl 140 mM in MilliQ water, salts from Synth) to remove free fungal cells, and macrophages were lysed with 0.1% Triton X-100 solution to recover phagocytosed fungi. 10-fold dilutions of the samples were plated on Sabouraud Dextrose Agar (BD) and incubated at 37 °C for 24 h. Recovered colonies were counted and results were expressed as log (CFU).

### Interaction between *C. albicans* yeast and J774 macrophages

J774 macrophages were plated on glass slides in 24-well plates (2.0 × 10^5^ cells/ml/well). Adherent monolayers were pre-incubated with PRM (100 μg/ml) or only with RPMI medium as untreated control for 1 h (1 ml/well). After this time, the supernatant was removed and *C. albicans* yeast cells were added to the adherent monolayers at a ratio of 1:1 (yeast: macrophage) and incubated for 1, 2 and 3 h. After that, supernatants were harvested for cytokine measurements as described above. Besides this, the same procedure was made in glass slides that were stained with commercial Giemsa (Instant Prov) and analyzed by optical microscopy (Additional file 1).

### Determination of MIC and MFC

Minimum inhibitory concentration (MIC) of PRM from *L. prolificans* against *C. albicans* was determined by broth microdilution performed for different concentrations (400–0.78 μg/ml) according to CLSI document M27-A3 (2008) [23]. In addition, itraconazole (Sigma–Aldrich, St Louis, MO, USA), was used as reference compound (8–0.015 μg/ml). Minimum Fungicidal concentration (MFC) was determined by sub-culturing an aliquot of 10 μl from each well that showed complete growth inhibition in Sabouraud agar medium without addition of PRM, and yeast growth was evaluated after 24 h at 37 °C. The MFC values were defined as the lowest concentration of PRM able to inhibit yeast.

### Statistical analysis

Statistical analyses were performed using GraphPad Prism version 5.00 for Windows (Graph-Pad Software, San Diego CA). Two-way ANOVA was used to compare differences between groups, and individual comparisons of groups were made using the Bonferoni test (Bonferoni posttest). A 90–95% confidence interval was determined in all experiments.

## Supplementary information

**Additional file 1. **PRM effect on macrophage – *Candida albicans* interaction.

## Data Availability

All datasets generated for this study are included in the manuscript.

## Abbreviations

CFU: Colony-Forming Units
LDH: Lactate dehydrogenase
HEp2: Larynx carcinoma cells
MIC: Minimum inhibitory concentration
MFC: Minimum Fungicidal concentration
PAMP: Pathogen-associated molecular pattern
PRRs: Pattern recognition receptor
PRM: Peptidorhamnomannan
PBMCs: Peripheral blood mononuclear cells
