# Supplementary material for: Peptidorhamnomannan from Lomentospora prolificans modulates the inflammatory response in macrophages infected with Candida albicans
Source: BMC Microbiol. 2020 Aug 6;20:245. doi: 10.1186/s12866-020-01931-3 (PMC7412847; doi:10.1186/s12866-020-01931-3)
Supplement: Supplementary file 1 — Additional file 1. PRM effect on macrophage – Candida albicans interaction. [file 12866_2020_1931_MOESM1_ESM.docx]

**PRM effect on macrophage – *Candida albicans* interaction**

J774 macrophages were plated on glass slides in 24-well plates (2.0 x10^5^ cells/ml/well). Adherent monolayers were pre-incubated with PRM (100 μg/ml) or only with RPMI medium as untreated control for 1h (1 ml/well) at 37°C. After this time, the supernatant was removed and the macrophage monolayer was rinsed with RPMI, *C. albicans* yeast cells were added to the adherent monolayers at a ratio of 1:1 (yeast : macrophage) and incubated for 1, 2 and 3h at 37°C. After that, glass slides were stained with commercial Giemsa (Instant Prov) and analyzed by optical microscopy. **Figure 1** shows the interaction between J774 macrophages and *C. albicans* yeast cells in different times of incubation and after 3h of incubation the macrophage seems to succumb yeast filamentation to hyphae after being phagocytosed by macrophages. However, macrophages pre-incubated with PRM seems to be more resistant in all times of incubation, and yeast seems to be less germinated after 3h of incubation (**Figure 2**). These results are in agreement with fungal viability assessed by determining the colony-forming units (CFU) that showed a less fungal burden when macrophages were primed with PRM.


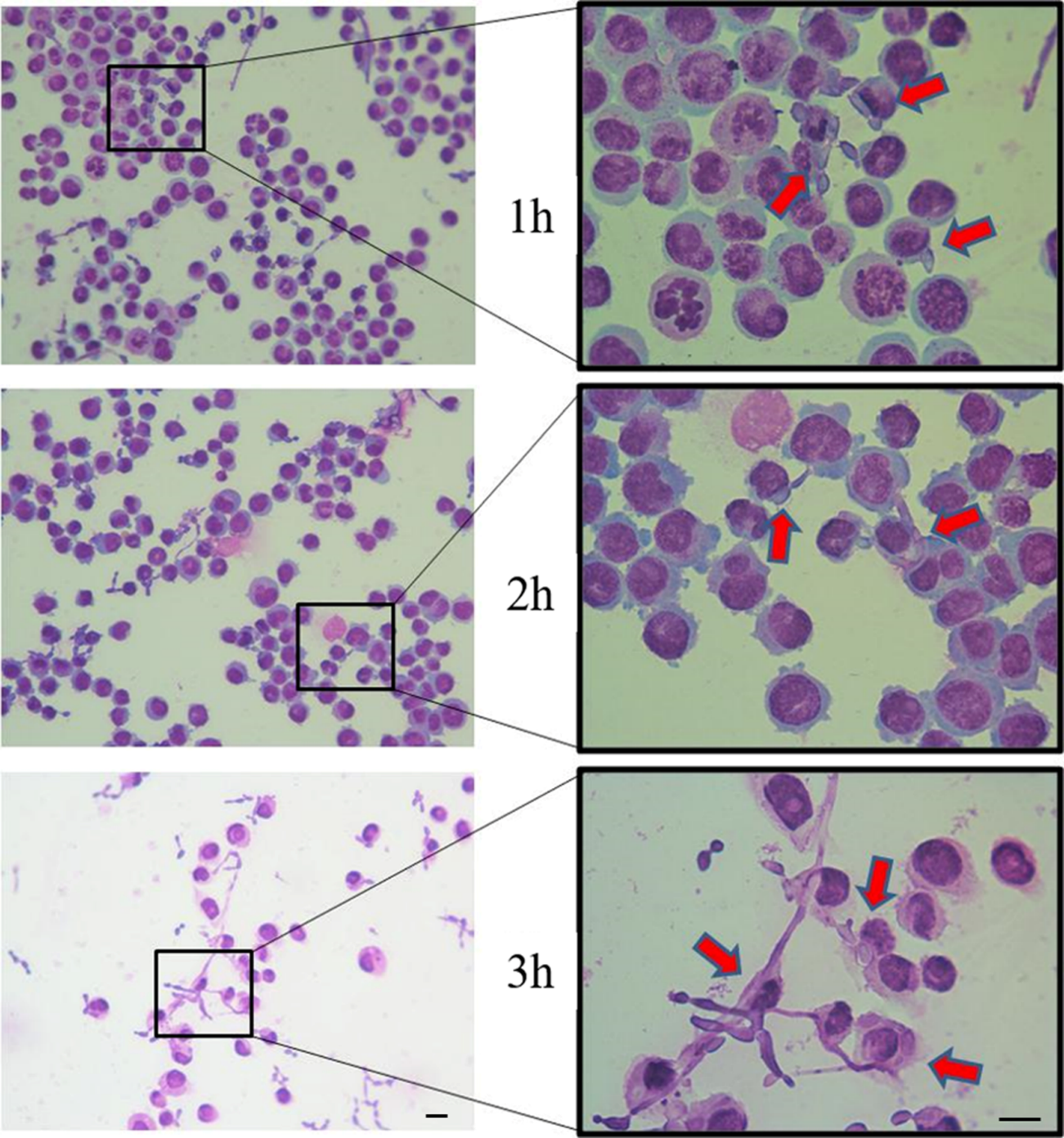
 Figure 1.

**Figure 1.** Light micrographs of *Candida albicans*-macrophage interaction at different times of incubation. J774 macrophages were incubated with *C. albicans* yeast cells (1:1 ratio) for 1 (a.), 2 (b.) and 3h (c.). Red arrows indicate yeast filamentation during incubation with macrophages. Bar = 20µm.

Figure 2.


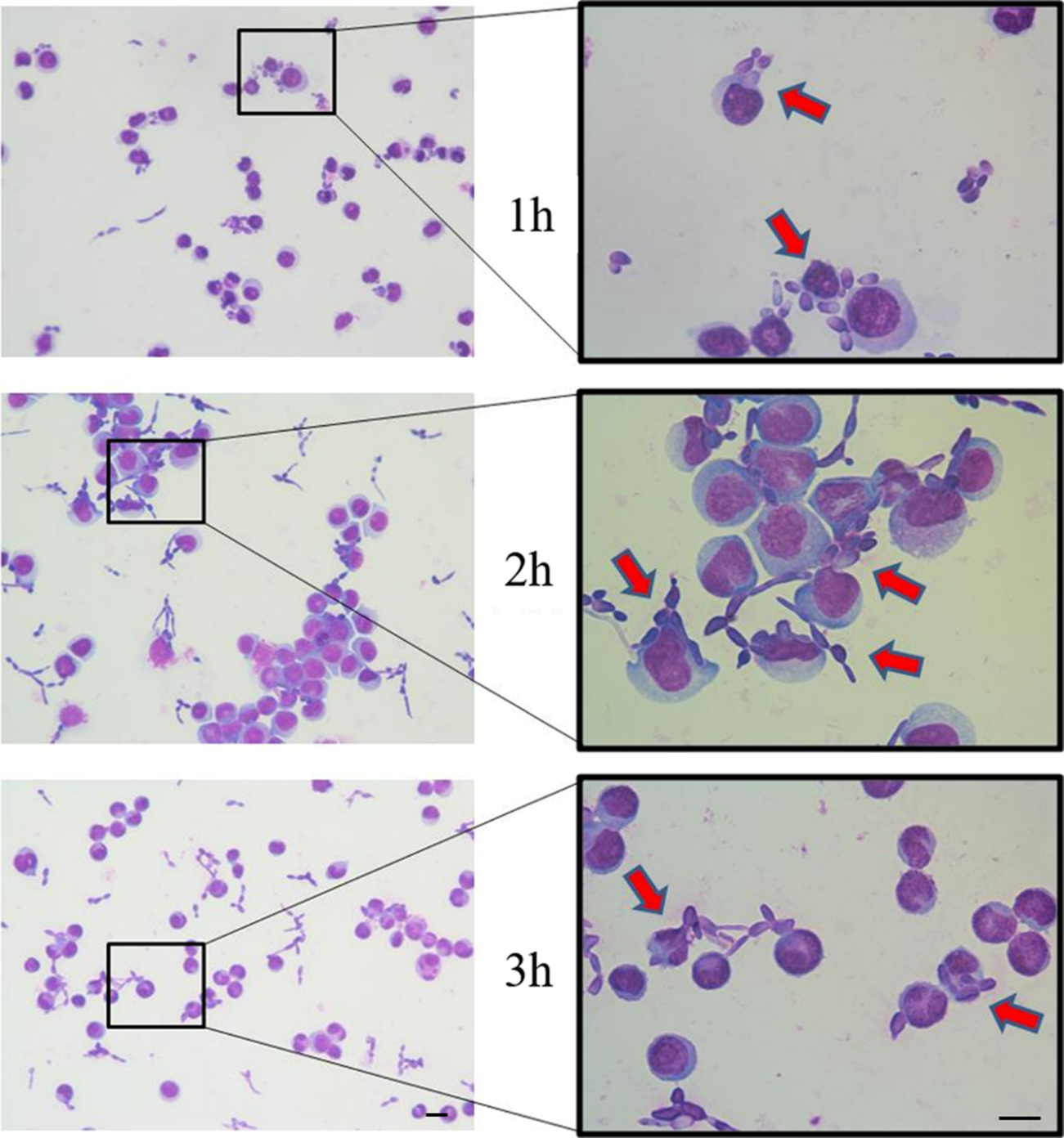


**Figure 2.** Light micrographs of *Candida albicans*-macrophage interaction at different times of incubation primed with PRM. J774 macrophages were incubated with PRM at 100 μg/ml for 1h. After this incubation, PRM was removed and the monolayer were washed, and incubated with *C. albicans* yeast cells (1:1 ratio) for 1 (a.), 2 (b.) and 3h (c.). Red arrows indicate yeast filamentation during incubation with macrophages that were primed with PRM. Bar = 20µm.
